# Elucidation of Phytochemicals Affecting Platelet Responsiveness in Dangguisu-san: Active Ingredient Prediction and Experimental Research Using Network Pharmacology

**DOI:** 10.3390/plants12051120

**Published:** 2023-03-02

**Authors:** Dong-Ha Lee, Hee Jae Kwak, Yonghee Shin, Sung Jin Kim, Ga Hee Lee, Il-Ho Park, Seung Hyun Kim, Ki Sung Kang

**Affiliations:** 1 Department of Biomedical Laboratory Science, Namseoul University, Cheonan 31020, Republic of Korea; 2 College of Pharmacy, Yonsei Institute of Pharmaceutical Science, Yonsei University, Incheon 21983, Republic of Korea; 3 College of Korean Medicine, Gachon University, Seongnam 13120, Republic of Korea; 4 College of Pharmacy, Sahmyook University, Seoul 01795, Republic of Korea

**Keywords:** phytochemicals, platelet, active ingredient, network pharmacology

## Abstract

Plant-derived phytochemicals are emerging as novel agents for protection against chronic disorders. Dangguisu-san is a herbal prescription to invigorate the blood and relieve pain. Among the numerous active constituents of Dangguisu-san, those expected to be effective at inhibiting platelet aggregation were predicted using a network pharmacological method, and their efficacy was experimentally demonstrated. All four identified chemical components, namely chrysoeriol, apigenin, luteolin, and sappanchalcone, suppressed the aggregation of platelets to a certain extent. However, we report, for the first time, that chrysoeriol acts as a strong inhibitor of platelet aggregation. Although additional in vivo studies are needed, among the complex constituents of herbal medicines, the components that exert an inhibitory effect on platelet aggregation were predicted using a network pharmacological method and experimentally confirmed with human platelets.

## 1. Introduction

Cardiovascular disease (CVD) is non-communicable and the principal cause of mortality and chronic disability worldwide [1]. Despite ongoing advances in surgical, medical, and interventional treatments, since 1990, approximately 17.9 million people globally have developed heart or circulatory disease each year [2,3]. The most common CVDs are coronary heart disease, peripheral arterial disease, stroke, and atrial fibrillation. Furthermore, the major contributing factor in most cases of cardiovascular disease and cerebrovascular disease (stroke) is atherosclerosis. CVD is caused by a narrowing of the walls of blood vessels, especially the arteries, due to the accumulation of plaque, which is composed of fatty deposits. To protect the body, platelets are activated by hemostasis and agglomerate to restore punctured blood vessels. Platelet activity increases when vessels are injured or when there is a change in blood flow or concentration in the vessels due to poor lifestyle habits, such as stress and lack of exercise. Therefore, as plaque formation continues, it eventually results in plaque rupture, blocking blood flow.

By suppressing the capacity of platelets to coagulate, antiplatelet drugs assist in preventing clot formation, which may block the vascular endothelium and lead to CVD or cerebrovascular disease [4]. Antiplatelet drugs, or platelet aggregation inhibitors, inhibit thrombus formation. When blood vessels are damaged, platelets attach to or aggregate in the damaged area, forming a lump. Therapeutic drugs modulate this process through various mechanisms of action, such as the inhibition of cyclooxygenase-1 (COX-1), PDE, ADP receptor antagonists, GP2b/3a inhibitors, and 5-HT2 receptor inhibitors. Aspirin, a cyclooxygenase (COX) inhibitor, is the most commonly used antiplatelet agent. Depending on the target proteins, other oral agents, such as ticlopidine (ADP receptor antagonist), clopidogrel (ADP receptor antagonist), dipyridamole (PDE inhibitor), or intravenous antiplatelet drugs, such as abciximab or eptifibatide, can be administered [4].

Salicin, a natural product in willow bark, is chemically similar to aspirin [5]. Aspirin or acetylsalicylic acid (ASA) is a nonsteroidal analgesic obtained by replacing the alcohol group of salicylic acid with an acetyl group. Salicylic acid is obtained by substituting a carboxylic acid for the beta-glucoside portion of salicin; ASA also has an analgesic effect. As a result, an acid–base relationship is created, which shows many improved effects compared with salicylic acid. ASA exists in its normal state in the acidic environment of the stomach, but in the relatively basic environment of the small intestine, the Lewis base and carboxyl group react to transform it into an ion (conjugate base) from which hydrogen is removed, making it easier to dissolve. Therefore, it does not dissolve well in the stomach but breaks down rapidly and efficiently in the small intestine, where it is absorbed [6]. In addition, since blood is relatively basic, ASA primarily remains in an ionized state in the blood, allowing it easy passage through the polar parts of cell membranes.

Aspirin activates its antiplatelet mechanism by acrylating key serine residues in the active site of the COX-1 isozyme, thereby inhibiting platelet COX activity and thromboxane-dependent platelet activation. COX-1, an important enzyme that produces prostanoids in arachidonic acid metabolism, is permanently blocked by aspirin, which converts arachidonic acid to an unstable intermediate prostaglandin G2 (PGG2). The subsequent metabolism of PGG2 leads to prostaglandin H2 synthesis via hydroperoxidase (HOX), which is finally converted to prostanoids by tissue-specific isomerases. Aspirin acts on COX-1 to inhibit the production of thromboxane A2 (TxA2) synthetase, thereby reducing TxA2-dependent platelet activation [7].

However, antithrombotic agents can interfere with blood coagulation, and thus, homeostasis, which can be fatal. In patients undergoing internal or traumatic surgery, or those with gastric ulceration or kidney disease, aspirin should be taken with caution due to its antithrombotic and anticoagulant properties. To explore alternatives, extensive research has been conducted to address the roles of phytochemicals in antiplatelet therapy related to CVD [8]. In vivo studies have revealed that the platelet inhibition effect of grape beverages in dogs [9] and monkeys [10] depends on nitric oxide (NO) production [11]. In addition, studies have demonstrated that grape juice consumption for 14 d decreases platelet aggregation and superoxide production and increases NO production in healthy volunteers [12]. Grape-derived polyphenols (flavonoids) inhibit platelet activity and polyphenols in vitro, which may decrease the cholesterol absorption capacity [13]. In addition, combinations of phytochemicals, whose chemical structures have been modified to mimic effective antiplatelet drugs, potentiate the effect of currently marketed antiplatelet drugs [14]. Therefore, phytochemicals can effectively replace or supplement antithrombotic agents.

In our experiment, the effects of various phytochemicals derived from Dangguisu-san, a herbal prescribed to invigorate the blood and relieve pain, on platelet responsiveness were investigated using a pharmacological network. Moreover, an in vitro experiment was conducted to verify the effects of chrysoeriol, apigenin, luteolin, and sappanchalcone, the constituents of Dangguisu-san. The results of this study could be utilized to aid the development of pharmaceuticals and health-functional foods to inhibit or treat platelet aggregation using chrysoeriol in the future.

## 2. Results

### 2.1. Network Pharmacology Analysis

#### 2.1.1. Collecting Chemical Ingredients and Selecting Expected Active Compounds (EAC)

A total of 119 compounds derived from Dangguisu-san were collected manually by searching databases. All these compounds were evaluated for drug-likeness (DL) and oral bioavailability (OB) using the quantitative estimate of drug-likeness (QED) method and Veber’s rule (Table S1). The QED of compounds was calculated using eight physicochemical properties, such as molecular weight (MW), the octanol-water partition coefficient (ALOGP), number of hydrogen bond acceptors (HBA), number of hydrogen bond donors (HBD), polar surface area (PSA), number of rotatable bonds (RTOB), number of aromatic rings (AROM), and number of structural alerts (ALERT). OB was judged on the basis of Veber’s rule: ROTB ≤ 10, sum of HAB and HBD ≤ 12, and PSA ≤140. The QED and OB cut-off values for selecting expected active compounds (EAC) were set as QED ≥ 0.3 and TRUE, respectively. Based on these cut-off values, 95 compounds were selected as the EAC (Table S2).

#### 2.1.2. Prediction of Target Genes and Identification of Potential Targets

**Figure 1:**
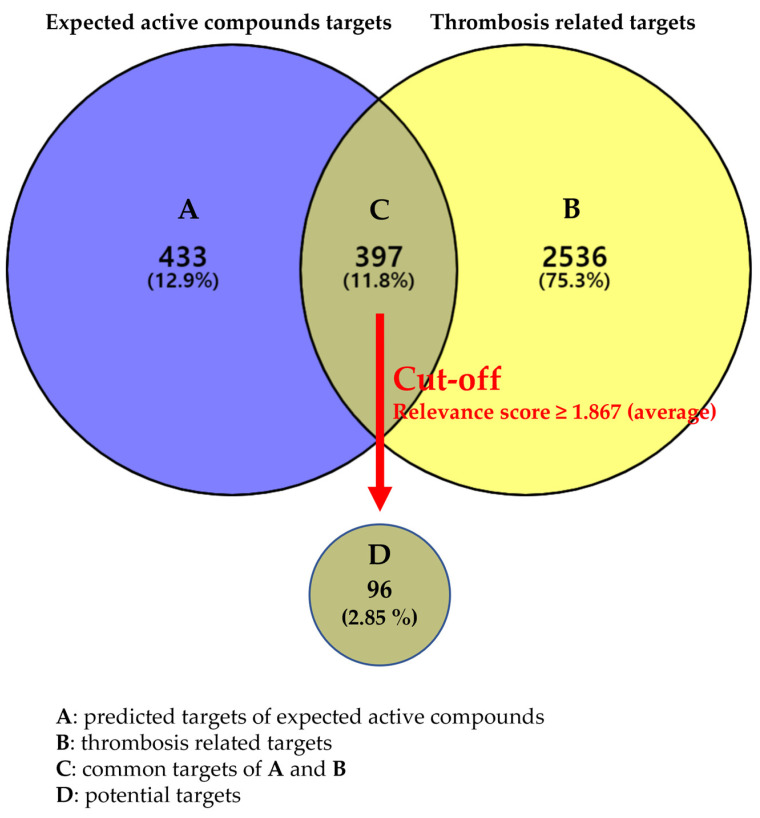
Venn diagram of EAC and disease-related targets.

The SwissTargetPrediction and GeneCards databases [15,16] were used to predict the targets of EAC and disease-related targets. After removing duplicates and false-positive targets, 397 genes intersected, and a disease relevance score (≥1.865) was used as the cut-off value to select potential targets. Consequently, 96 potential targets were identified (Figure 1, Table S3).

#### 2.1.3. Analysis of Protein–Protein Interaction (PPI) Networks of Potential Targets and Key Targets

**Figure 2:**
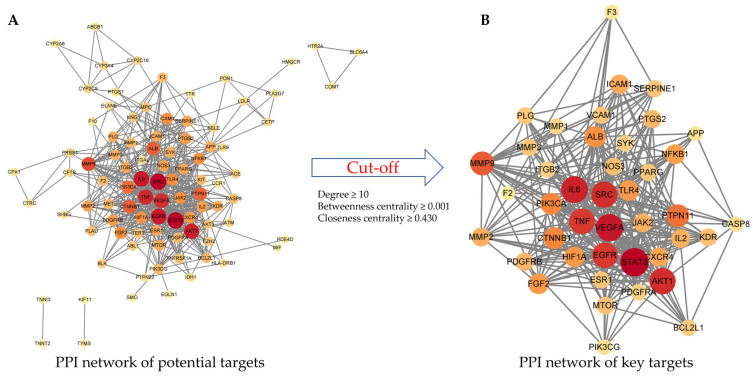
PPI networks: (**A**) PPI network of potential targets; (**B**) PPI network of key targets.

The potential targets were uploaded to the STRING database [17] to obtain the PPI of potential targets, and the interaction data was imported into Cytoscape (Ver. 3.9.1) [18] to construct and analyze the PPI network (Figure 2A). Topological network analysis was performed using three analytical parameters: degree (degree centrality), betweenness centrality, and closeness centrality. These parameters indicate the importance of nodes within the network [19,20,21]. Target genes satisfying specific conditions (degree ≥10, betweenness centrality ≥ 0.001, and closeness centrality ≥ 0.430) were selected as key targets (Table 1). The PPI network of the key targets consisted of 42 target nodes and 323 edges (Figure 2B). Among them, STAT3 (signal transducer and activator of transcription 3) showed the highest degree value. STAT3 is a transcription factor activated by cytokine-induced intracellular signals that regulates the transcription of various genes required for plate production [22]. In addition, vascular endothelial growth factor A, interleukin 6 (IL-6), AKT serine/threonine kinase 1 (AKT1), and proto-oncogene tyrosine protein kinase Src (SRC) showed stronger interactions with other genes (degree ≥ 25). These genes regulate factors related to thrombosis [23,24,25].

#### 2.1.4. Kyoto Encyclopedia Genes and Genomes (KEGG) Signaling Pathway Enrichment Analysis

**Figure 3:**
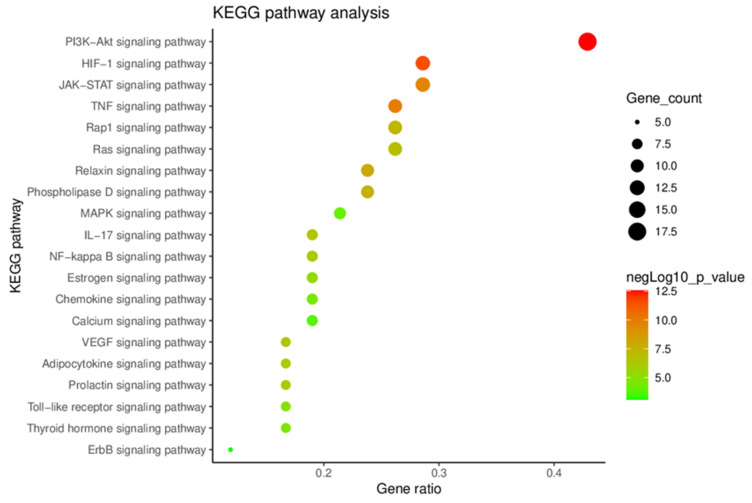
A bubble chart of KEGG signaling pathway enrichment analysis.

A KEGG signaling pathway enrichment analysis of key targets was performed using the DAVID [26] to identify related signaling pathways (Figure 3). In the KEGG signaling pathway analysis, 110 KEGG terms were acquired, and the top 20 terms were selected based on the *p*-value. As shown in Figure 3, the PI3K-Akt signaling pathway showed the highest *p*-value and gene ratio. According to previous studies, it is highly correlated with the regulation of platelet activation and thrombosis [27,28,29,30]. Therefore, this result suggests that the PI3K-Akt signaling pathway may be the main antithrombotic mechanism of Dangguisu-san.

#### 2.1.5. Analysis of Integrated Herbs-EAC-Key Targets-Pathways (H-C-T-P) Network

**Figure 4:**
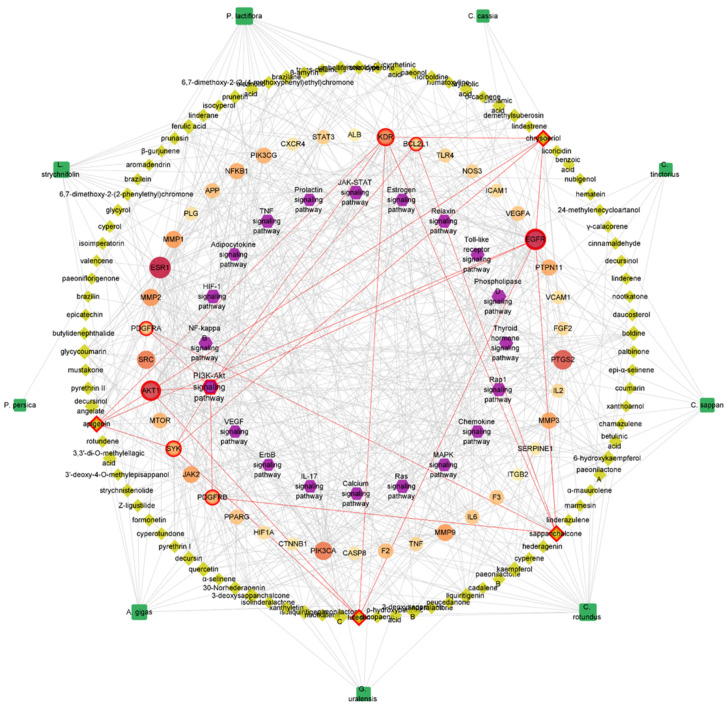
Integrated H-C-T-P network.

An integrated H-C-T-P network was constructed for the complete interpretation of the network pharmacology analysis. The H-C-T-P network consisted of 165 nodes (9 herbs (green), 94 EAC (yellow), 42 key targets (reddish), and 20 pathways (purple)) and 621 edges (Figure 4), and a topological network analysis was performed. The topological analysis was evaluated with three centrality indicators: degree, betweenness and closeness centrality. These parameters represent the importance of a node within the network [31]. In particular, since degree means the number of nodes connected with other nodes, it is the most intuitive factor to evaluate importance. Therefore, the key components and the main signaling pathway were selected mainly by reflecting the degree value. Among the EAC nodes, four compounds, apigenin, chrysoeriol, luteolin, and sappanchalcone, showed the highest degree value and were selected as key components (Table 2). Furthermore, the PI3K-Akt signaling pathway exhibited a degree value of 18, which was the highest value of the 20 signaling pathways. The key components and the PI3K-Akt signaling pathway were commonly linked to key targets of SYK, EGFR, KDR, AKT1, BCL2L1, PDGFRA, and PDGFRB. Among them, EGFR and KDR are connected to all the key components. The GPCR-mediated transactivation of EGFR and KDR induces downstream signaling such as PI3K/Akt [32,33]. Activated AKT plays a crucial role in platelet activity as a downstream effector of PI3K [24]. Therefore, these results suggest that the key components and mechanism of action deduced by network pharmacology analysis contribute sufficiently to the antithrombotic activity of Dangguisu-san.

### 2.2. Key Component Chemical Profiling Analysis

High-performance liquid chromatography/quadrupole-time-of-flight tandem mass spectrometry (HPLC-Q-TOF-MS) analysis was performed to verify the prediction of the key components highlighted in the network pharmacology analysis. The extract of Dangguisu-san powder was analyzed using optimized HPLC-Q-TOF-MS conditions. The total compound chromatogram (TCC) of the negative ion mode is shown in Figure S1. Characterization of all the peaks in the first-order mass spectrum presented the charge carrier of [M-H]^-^ in a negative ion mode. By comparing m/z values, four key components were identified (Table 3).

### 2.3. Effects of Dangguisu-san and Its Components on Agonist-Induced Platelet Aggregation

**Figure 5:**
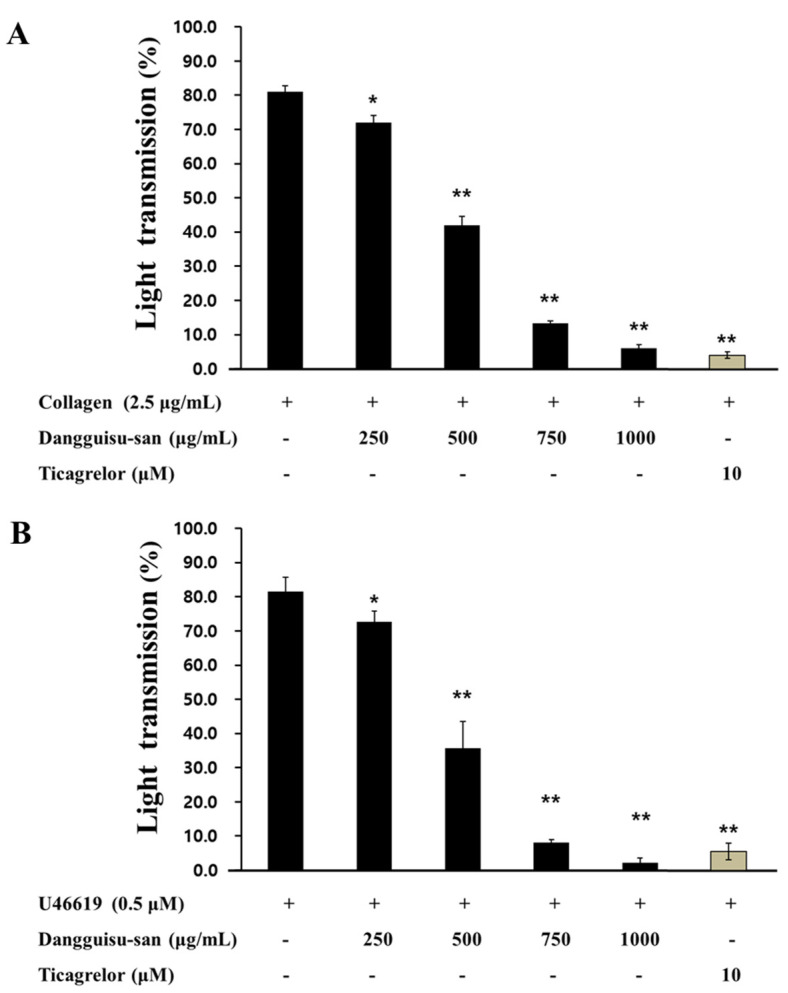
Effects of Dangguisu-san and positive control on agonist-induced platelet aggregation. (**A**) Effect of Dangguisu-san on collagen-induced human platelet aggregation (**B**) Effect of Dangguisu-san on U46619-induced human platelet aggregation. The results are shown as mean ± SD (*n* = 4). * *p* < 0.05, ** *p* < 0.001 in comparison to the agonist-induced vehicle control.

**Figure 6:**
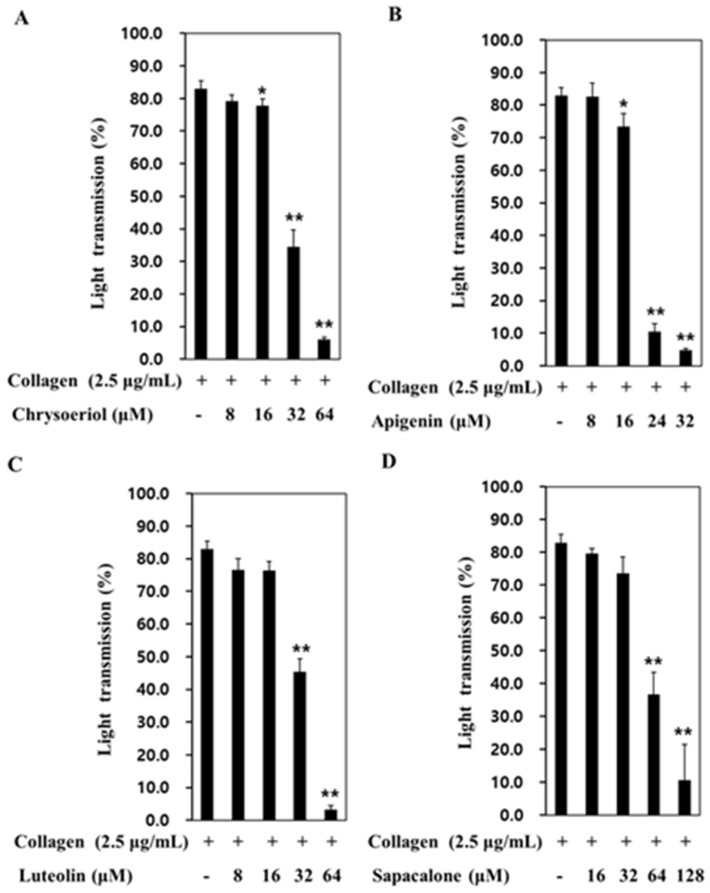
Effects of four key components on agonist-induced platelet aggregation. (**A**) Chrysoeriol. (**B**) Apigenin. (**C**) Luteolin. (**D**) Sappanchalcone. The results are shown as mean ± SD (*n* = 4). * *p* < 0.05, ** *p* < 0.001 in comparison to the agonist-induced vehicle control.

We confirmed that extracts of Dangguisu-san had a concentration-dependent inhibitory effect on collagen and U46619-induced human platelet aggregation (Figure 5). In addition, it was confirmed that the major components of Dangguisu-san had an effect of inhibiting platelet aggregation. In the platelets mixed with 2.5 μg/mL collagen and 0.1% DMSO to start aggregation, the rate of aggregation came to 83.0 ± 2.4% (vehicle control), which was not significantly different from that when induced without 0.1% DMSO. The results revealed that aggregation was suppressed by all four key components (Figure 6). Comparing the four components, the half-maximal inhibitory concentrations (IC50) of chrysoeriol, apigenin, luteolin, and sappanchalcone were 27.32 µM, 18.01 µM, 27.97 µM, and 57.12 µM, respectively (Figure 6). These results reveal, for the first time, that chrysoeriol acts as a strong inhibitor of platelet aggregation. Its effect is similar to or stronger than that of apigenin, luteolin, or sappanchalcone, which are the components already known to have an effect on platelet aggregation. Since chrysoeriol is structurally similar to apigenin and luteolin, we expected that it would suppress platelet aggregation by inhibiting the thromboxane A2 (TxA2) receptor, and confirmed that the four substances strongly inhibited TxA2 production in agonist-induced platelets (Table 4).

## 3. Discussion

Network pharmacology analysis is a useful method that can scientifically interpret the traditional activities of herbal medicines. In this study, a network pharmacological approach was applied to identify the bioactive components of Dangguisu-san against anti-platelet aggregation activity. Dangguisu-san is the most commonly prescribed herbal medicine covering blood stasis in the traditional medicine of China, Japan, and Korea [34]. According to previous studies, the constituent herbs of Dangguisu-san, such as *Angelica gigas* (Angelica Radix), *Paeonia lactiflora* (Paeoniae Radix), and *Carthamus tinctorius* (Carthami Flos) showed anti-platelet aggregation [35,36,37]. However, Dangguisu-san, which is prepared by extracting several herbs together, including those mentioned above, is a different extract from the one made by mixing each herb’s extract. Therefore, it does not guarantee that a herbal ingredient contributes to the bioactivity of Dangguisu-san although it has an antiplatelet activity. In that sense, network pharmacology can efficiently assist an exploration of which compounds derived from the herbal ingredients that make up Dangguisu-san contribute to antiplatelet activity.

Based on the results of network pharmacology, the four key components of the predictive antithrombosis effect of Dangguisu-san were revealed as apigenin, chrysoeriol, luteolin, and sappanchalcone. Apigenin and luteolin are supposed to be derived from *C. tinctorius*. Chrysoeriol and sappanchalcone are reportedly from *C. rotundus* and *C. sappan*, respectively. Although the previously reported activities of these compounds or herbs containing them do not exactly correspond to the antiplatelet activity of Dangguisu-san observed in the present study, it was found that their known activities may affect the antiplatelet activity of Dangguisu-san [38,39,40,41]. Apigenin modulated platelet adhesion and thrombus formation by suppressing the arachidonic acid pathway, while luteolin inhibited platelet function by binding to the thromboxane A2 receptor [38,39]. In addition, chrysoeriol and sappanchalcone showed anti-platelet aggregation activity [40,41]. Furthermore, these compounds were predicted to target both EGFR and KDR genes which are known to induce the phosphorylation of AKT and the AKT-mediated activation of PI3K, which has been implicated in playing an important role in platelet activation. Consequently, these results suggest that the four key components and PI3K-Akt signaling pathway are highly relevant to the antithrombotic effect of Dangguisu-san.

In addition, the anti-platelet activities of other EAC and the herbal components of Dangguisu-san have been reported in previous studies. For example, ferulic acid (derived from *A. gigas*) inhibited platelet-induced clot retraction, decreased the secretion of the granule constituent, and downregulated α_IIb_β_3_/FIB/AKT signaling [42]. Brazilin (derived from *C. sappan*) showed an anti-platelet aggregation effect by inhibiting phospholipase A_2_ and [Ca^2+^] elevation [43]. *C. rotundus* EtOH extract and its derived sesquiterpenoid, nootkatone, showed dose-dependent inhibitory effects on platelet aggregation in vitro and in vivo [44]. Moreover, the extract of *C. tinctorius* inhibited ADP-induced human platelet aggregation [45]. Based on the evidence, we can assume that Dangguisu-san exhibits an antithrombosis effect through the multi-target effects of the key components identified in this study and other EAC derived from Dangguisu-san. A network pharmacology analysis could efficiently suggest the key contributors in Dangguisu-san and their antithrombotic-activity-related targets. Moreover, the analysis of the networks among these targets revealed the biological pathways involved in the mechanism of action for Dangguisu-san.

HPLC-Q-TOF-MS is a widely used tool for the identification of chemical components in the extract of natural products [46,47]. Using HPLC-Q-TOF-MS, the key components’ chemical profiling was performed in order to verify the presence of the predicted four key components, since these substances have been generally reported from an organic solvent extract of a single herb. Thus, it is necessary to experimentally validate whether these compounds are actually contained in the extract of Dangguisu-san prepared by extracting several herbs together. According to the analysis result, all the key components were identified in the extract of Dangguisu-san. This result indicates that the predicted key components from the network pharmacology are indeed present and may contribute to the antithrombotic effect of Dangguisu-san.

In the meantime, flavonoids have attracted attention for medicinal use because they have many beneficial effects on cardiovascular health [48]. Some flavonoids inhibited platelet aggregation, and irrespective of their potential effects on signaling pathways, the binding of flavonoids to cellular receptors has been postulated as a possible mechanism of their antithrombotic activity [49,50]. Red wine inhibits the binding of platelet-derived growth factor (PDGF) to receptors on vascular smooth muscle cells [51]. In addition, genistein inhibited the binding of the TxA2-analog [3H]-U46619 to washed platelets [52,53]. Additionally, isoflavones inhibited the progression of atherosclerosis in vivo in a cholesterol-fed rabbit model [54].

In this study, among the four substances derived from Dangguisu-san which strongly inhibit platelet aggregation, the order in which they inhibit is chrysoeriol, apigenin, luteolin, and sapacalone. A previous study reported that flavonoids such as apigenin and luteolin interacted with TxA2 receptors with high affinity, and the tight binding of flavonoids to TxA2 receptors affected the steric and electrostatic properties, reducing the affinity for TxA2 receptors, thereby reducing collagen-induced platelet aggregation [39]. All four substances isolated from Dangguisu-san also reduced the extent of TxA2 production in collagen-stimulated platelets in our results. There is clinical evidence that inhibiting the TxA2 signaling pathway in platelets can treat or prevent thrombotic diseases [55]. Indeed, aspirin, which inhibits TxA2 synthesis, is widely used in recurrent myocardial infarction and thromboembolic stroke. In addition, it has been suggested that flavonoids with high aggregation inhibitory effects on the TxA2 receptor may inhibit the collagen signaling pathway [56]. Our results indicate that the active ingredients of Dangguisu-san are substances that inhibit TxA2 production more than the same concentration of aspirin, and it might be worth using instead them of aspirin applications.

Further research is needed on the antiplatelet action mechanism of the components of Dangguisu-san. Nonetheless, the antiplatelet effect of chrysoeriol, an extract from Dangguisu-san which inhibits human platelet aggregation in a concentration-dependent manner, was confirmed for the first time, so the novelty of this study should be recognized. Therefore, we suggest that the application of Dangguisu-san might be beneficial for cardiovascular diseases such as thrombosis, atherosclerosis, and stroke by inhibiting platelet aggregation through regulation of the TxA2 signaling pathway.

## 4. Materials and Methods

### 4.1. Network Pharmacology Analysis

#### 4.1.1. Collecting Chemical Components of Dangguisu-san and Selecting EAC

The chemical components of Dangguisu-san were manually collected through databases, including National Herbal Medicine Information (NHMI, https://nifds.go.kr/nhmi/main.do (accessed on 18 May 2022)), Oriental Medicine Advanced Searching Integrated System (OASIS, https://oasis.kiom.re.kr/ (accessed on 18 May 2022)) and PubChem (https://pubchem.ncbi.nlm.nih.gov/ (accessed on 18 May 2022)) [57,58,59]. All ingredients were evaluated for their DL and OB using the QED method and Veber’s rule [60,61]. The physicochemical properties of the components were obtained from the SwissADME (https://www.swissadme.ch/ (accessed on 18 May 2022)) [62]. All the properties were introduced into the QED function to calculate the QED value, then two cut-off values (QED ≥ 0.3 and OB = TRUE) were set to select the EAC.

#### 4.1.2. Acquisition of EAC and Disease-Related Targets

Canonical SMILES codes of the EAC were obtained from PubChem (https://pubchem.ncbi.nlm.nih.gov/ (accessed on 18 May 2022)) and uploaded to SwissTargetPrediction (http://www.swisstargetprediction.ch/ (accessed on 18 May 2022)) [15] to obtain the predicted targets. Disease-related targets were detected using GeneCards (https://www.genecards.org/ (accessed on 18 May 2022)) [16]. “thrombosis” was used as a keyword for searching for the disease-related targets.

#### 4.1.3. Selection of Potential Targets

Potential targets were selected as follows: (1) duplicates and false-positive targets of the EAC were removed, (2) targets were compared with disease-related targets to obtain common targets, (3) a disease relevance score (≥1.865 (average)) was set as the cut-off value to screen potential targets. Specific protein class information of the potential targets was retrieved using the DisGeNET database (https://www.disgenet.org/search (accessed on 18 May 2022)) [63].

#### 4.1.4. Construction and Analysis of the PPI Network and Selection of Key Targets

The STRING database was used to analyze the PPI of potential targets (https://string-db.org/ (accessed on 18 May 2022)) [17]. The analysis parameters were set as follows: the required score was a high confidence (0.700) and a medium false discovery rate (FDR) stringency (5%). The analytical data were imported into Cytoscape (ver. 3.9.0) to construct and analyze the PPI network of the potential targets. Topological network analytical parameters, degree, betweenness centrality, and closeness centrality were used to estimate the nodes in the network. Key targets were selected from the potential targets based on the topological analysis results.

#### 4.1.5. Analysis of KEGG Signaling Pathway

KEGG signaling pathway enrichment analysis of the key targets was conducted using the DAVID database (https://david.ncifcrf.gov/home.jsp (accessed on 18 May 2022)) [26]. The false discovery rate (FDR) error control method (FDR < 0.05) was used to correct the *p*-value, and *p* of <0.05 was set as a threshold value to obtain signaling pathways. The KEGG pathway enrichment analysis result was visualized using ImageGP (http://www.ehbio.com/ImageGP (accessed on 18 May 2022)).

#### 4.1.6. Construction of Integrated Herbs-EAC-Key Targets-Pathways (H-C-T-P) Network

An integrated network of herbs, EACs, key targets, and signaling pathways was constructed and analyzed using the Cytoscape software.

### 4.2. Key Component Chemical Profiling Using HPLC-Q-TOF-MS

Dangguisu-san powder (10.0 g) was dissolved in 100 mL methanol (100%, *v*/*v*) and subjected to ultrasonic extraction at room temperature for 60 min. The extract was filtered through qualitative filter paper (5–8 µm, 150 mm) and concentrated under reduced pressure until dry. The dried samples were dissolved in methanol (100%, *v*/*v*) to prepare 10 mg/mL of a final concentration. HPLC analysis was performed using an Agilent 1290 HPLC system (Agilent Technologies, Santa Clara, CA, USA) equipped with a 1290 Infinity Binary Pump, autosampler, and photodiode array (PDA) detector. Separation was performed on a YMC-Pack Pro C18 RS (2.0 mm i.d. × 150 mm, 3.0 µm). The mobile phase consisted of H_2_O (containing 0.1% formic acid; solvent A) and CH3CN (containing 0.1% formic acid; solvent B) with the following gradient elution profile: 0.00–20.00 min, 10–100% B; 20.10–25.00 min, 100% B; 25.10–30.00 min, 10% B. The flow rate was set at 0.300 mL/min and the injection volume was 10.00 µL. Mass spectrometry was performed on an Agilent 6530 Q-TOF mass spectrometer (Agilent Technologies, Santa Clara, CA, USA) equipped with an electrospray ionization (ESI) interface and analyzed in the negative mode. All MS data were acquired using the MassHunter Data Acquisition Software and the “Find by Formula” function was used to detect the four key components.

### 4.3. Reagents and Chemcicals

Dangguisu-san was manufactured by Hanpoong Pharm & Foods Co., Ltd. (Jeonju, Republic of Korea). HPLC grade solvents were purchased from Burdick & Jackson (Muskegon, MI, USA) and J.T. Baker (Chemical Co., Philipsburg, NJ, USA). The 4 key components were purchased from ChemFaces^®^ (Wohan, China), and were ≥ 98% pure.

### 4.4. Preparation of Human Washed Platelets

Korean Red Cross Blood Center (Suwon, Republic of Korea) supplied the human platelet-rich plasma (PRP). PRP was centrifuged at 1300× *g* for 10 min to collect platelets. Then, it was washed with a buffer (pH 6.9, 2.7 mM KCl, 138 mM NaCl, 12 mM NaHCO_3_, 0.36 mM NaH_2_PO_4_, 1 mM Na_2_EDTA, and 5.5 mM glucose) twice. A suspension buffer (pH 7.4, 2.7 mM KCl, 138 mM NaCl, 12 mM NaHCO_3_, 0.49 mM MgCl_2_, 0.36 mM NaH_2_PO_4_, 5.5 mM glucose, 0.25% gelatin) as used to suspend the platelets (final concentration of 10^8^ cells/mL). Platelet aggregation was avoided by performing all procedures at 25 °C, and the Namseoul University Institutional Review Board (1041479-HR-201803-003) approved this experiment.

### 4.5. Platelet Aggregation Measurement

Incubation of the suspended platelets (10^8^ cells/mL) was carried out while adding candidate components at different concentrations for 3 min and at 37 °C. Then, 2 mM CaCl_2_ was added as well as collagen (2.5 μg/mL) for stimulation at a duration of 5 min. The aggregometer (Chrono-Log Co., Havertown, PA, USA) measured the experiment with a stirring speed of 1000 rpm, and the rate of aggregation was calculated with an increase in the light transmittance. The suspension permeability of a 0% suspension buffer was used as the reference value. The candidate components were dissolved with a concentration of 0.1% dimethyl sulfoxide (DMSO), and all tests were performed by adding the same dose of DMSO.

### 4.6. TxB_2_ Production Measurement

For 3 min, the incubation of the suspended platelets (10^8^ cells/mL) occurred at 37 °C while adding candidate components, and then 2 mM CaCl_2_ was added with collagen (2.5 μg/mL) for stimulation for a duration of 5 min. A synergy HT multi-reader (BioTek Instruments, Winooski, VT, USA) measured TxB_2_ (a stable TxA_2_ metabolite) production with the use of the TxB_2_ EIA kit.

## 5. Conclusions

This is the first study on the effects of phytochemicals derived from Dangguisu-san on platelet responsiveness using a network pharmacological method. The computational analyses demonstrated that chrysoeriol, apigenin, luteolin, and sappanchalcone may be the key components of Dangguisu-san conferring therapeutic effects against thrombosis. All of these four identified chemical components suppressed the aggregation of platelets to a certain extent. Among the active constituents of Dangguisu-san, all four identified chemical components, chrysoeriol, apigenin, luteolin, and sappanchalcone, suppressed the aggregation of platelets dose-dependently, and reduced TxA_2_ production in agonist-induced platelets. Among them, we report for the first time that chrysoeriol acts as a strong inhibitor of platelet aggregation. In this study, among the complex constituents of Dangguisu-san, the components which exert an inhibitory effect on platelet aggregation were predicted through a network pharmacological method and experimentally confirmed with human platelets. Although additional experimental validation for the mechanisms of action is needed in further study, we suggest that Dangguisu-san and its effective constituents are valuable as antiplatelet agents.

## Figures and Tables

**Table 1:** Key targets based on PPI network topological analysis.

Target Gene	Degree	Betweenness Centrality	Closeness Centrality	Relevance Score
STAT3	31	0.059	0.804	3.767
VEGFA	29	0.058	0.774	7.895
IL6	28	0.067	0.759	8.536
SRC	27	0.078	0.745	2.834
AKT1	27	0.041	0.745	11.390
EGFR	26	0.040	0.732	1.950
TNF	26	0.051	0.732	7.125
MMP9	23	0.026	0.695	3.936
PTPN11	21	0.019	0.672	2.467
CTNNB1	19	0.018	0.651	4.002
PIK3CA	19	0.014	0.641	9.507
ALB	18	0.028	0.641	7.226
HIF1A	17	0.009	0.631	2.834
FGF2	17	0.009	0.631	3.380
TLR4	17	0.012	0.631	7.630
NFKB1	16	0.017	0.621	2.011
CXCR4	15	0.016	0.612	2.595
ICAM1	15	0.011	0.612	5.260
IL2	14	0.006	0.603	2.973
MMP2	14	0.007	0.603	3.102
JAK2	14	0.008	0.603	36.781
PDGFRB	13	0.004	0.594	2.117
PTGS2	13	0.003	0.594	6.734
KDR	12	0.003	0.577	2.177
MTOR	12	0.006	0.569	2.209
NOS3	12	0.006	0.586	7.956
PPARG	11	0.003	0.577	2.515
ESR1	11	0.002	0.554	3.424
MMP3	11	0.003	0.569	3.432
VCAM1	11	0.007	0.577	4.597
PLG	11	0.005	0.562	12.016
SERPINE1	11	0.008	0.562	21.828
SYK	10	0.005	0.562	2.066
BCL2L1	10	0.003	0.569	2.575
ITGB2	10	0.006	0.569	2.812
PDGFRA	10	0.001	0.569	5.720
MMP1	9	0.000	0.539	2.561
CASP8	8	0.002	0.554	2.362
PIK3CG	8	0.002	0.519	3.213
APP	7	0.003	0.539	2.270
F3	7	0.002	0.506	23.179
F2	6	0.005	0.526	58.855

**Table 2:** List of key components of Dangguisu-san from the H-C-T-P network analysis.

Key Components	Structure	Formula	Degree
Apigenin	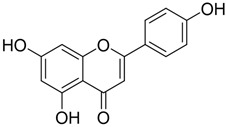	C_15_H_10_O_5_	14
Chrysoeriol	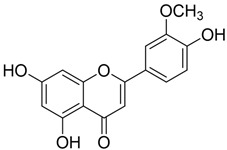	C_16_H_12_O_5_	14
Luteolin	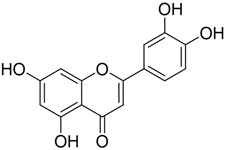	C_15_H_10_O_5_	14
Sappanchalcone	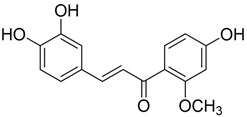	C_16_H_14_O_5_	14

**Table 3:** List of the key components detected in Dangguisu-san using HPLC-Q-TOF-MS analysis.

Key Components	Retention Time (min)	Observed m/z	Calculated m/z	Error ppm
Apigenin	13.924	279.0448 [M-H]^−^	269.0450	−2.47
Chrysoeriol	6.824	299.0559 [M-H]^−^	299.0556	−0.8
Luteoin	8.015	285.0410 [M-H]^−^	285.0399	2.19
Sappanchalcone	7.373	285.0842 [M-H]^−^	285.0763	0.35

**Table 4:** Effects of four key components on TxA2 production in human platelets.

	TxA2 Production in 10^8^ Platelets/mL
Intact cell	1.12 ± 0.21
Collagen 2.5 µg/mL	120.09 ± 11.97
Chrysoeriol 32 µM + collagen 2.5 µg/mL	28.23 ± 2.36 *
Apigenin 32 µM + collagen 2.5 µg/mL	23.97 ± 3.28 *
Luteoin 32 µM + collagen 2.5 µg/mL	29.53 ± 6.55 *
Sappanchalcone 32 µM + collagen 2.5 µg/mL	74.35 ± 11.81 *
Aspirin 32 µM + collagen 2.5 µg/mL	75.84 ± 12.06 *

* *p* < 0.05 in comparison to the agonist-induced vehicle control.

## Data Availability

Data are contained in manuscript.

## Supplementary Materials

The following supporting information can be downloaded at: https://www.mdpi.com/article/10.3390/plants12051120/s1, Figure S1: HPLC-Q-TOF-MS chromatogram of Dangguisu-san in negative mode; Table S1: List of physicochemical properties, QED and OB of Dangguisu-san derived compounds; Table S2: List of expected active compounds; Table S3: List of potential targets.
