# Supplementary material for: Elucidation of Phytochemicals Affecting Platelet Responsiveness in Dangguisu-san: Active Ingredient Prediction and Experimental Research Using Network Pharmacology
Source: Plants (Basel). 2023 Mar 2;12(5):1120. doi: 10.3390/plants12051120 (PMC10005453; doi:10.3390/plants12051120)
Supplement: Supplementary file 1 [file plants-12-01120-s001.zip › plants-2157200-supplementary.pdf]

**Supplementary data**

# Elucidation of Phytochemicals Affecting Platelet Responsiveness in Dangguisu-san: Active ingredient prediction and experimental research using network pharmacology

Figure S1. HPLC-Q-TOF-MS chromatogram of Dangguisu-san in negative mode

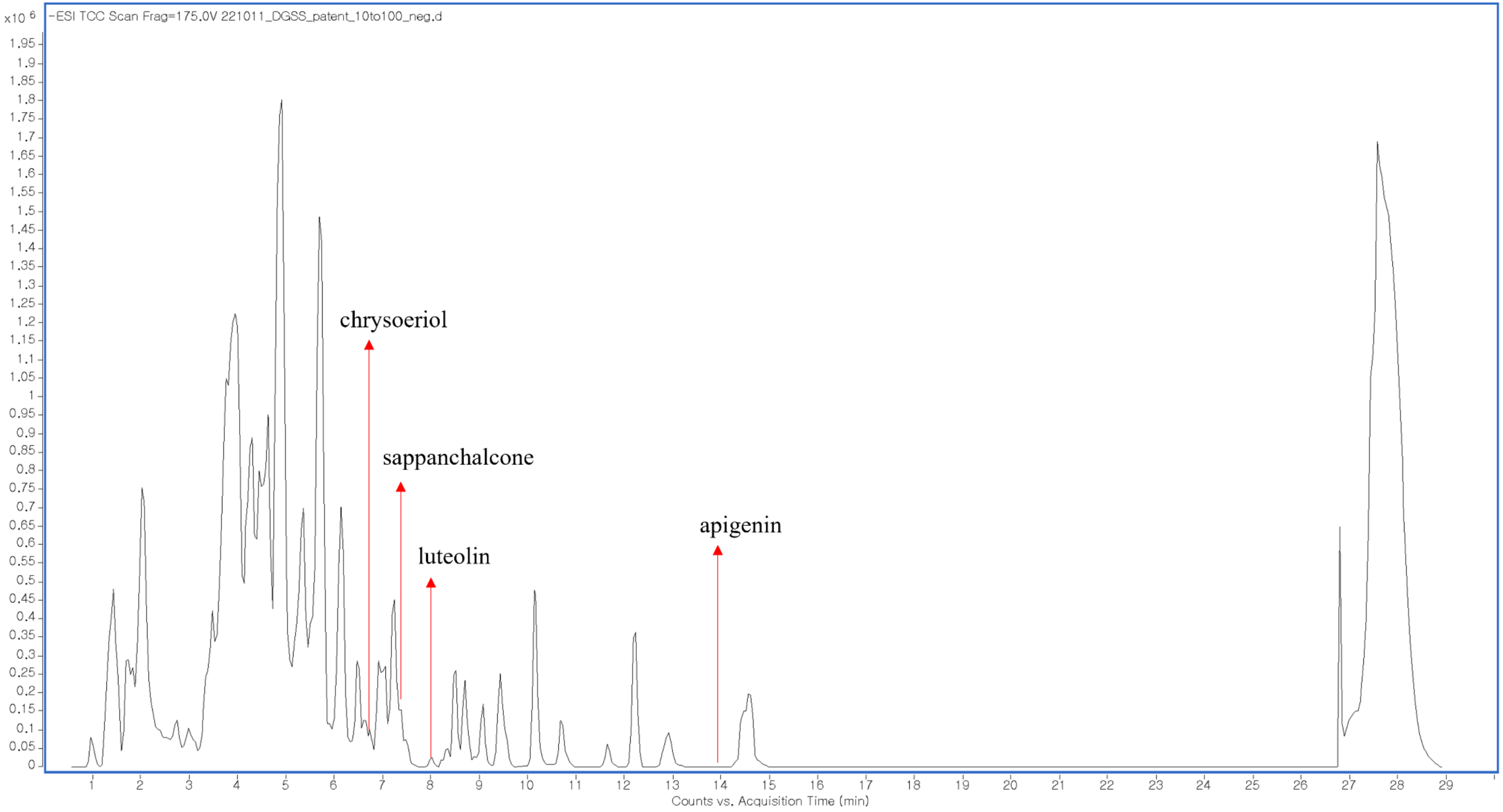

Table S1. List of physicochemical properties, QED and OB of Dangguisu-san derived compounds

| No. | Compound Name | MW | ALOGP | HBA | HBD | PSA | ROTB | AROM | ALERTS | QED | OB |
|---|---|---|---|---|---|---|---|---|---|---|---|
| | | | | | ***Angelica gigas*** | | | | | | |
| 1 | nodakenin | 408.40 | 0.67 | 9 | 4 | 138.82 | 4 | 2 | 1 | 0.538 | FALSE |
| 2 | decursin | 328.36 | 4.06 | 5 | 0 | 65.74 | 3 | 2 | 2 | 0.628 | TRUE |
| 3 | decursinol angelate | 328.36 | 4.06 | 5 | 0 | 65.74 | 3 | 2 | 2 | 0.628 | TRUE |
| 4 | isoimperatorin | 270.29 | 3.99 | 4 | 0 | 52.58 | 3 | 2 | 2 | 0.625 | TRUE |
| 5 | ferulic acid | 194.18 | 1.26 | 4 | 2 | 66.76 | 3 | 1 | 1 | 0.711 | TRUE |
| 6 | umbelliferone | 162.14 | 1.97 | 3 | 1 | 50.44 | 0 | 2 | 1 | 0.602 | TRUE |
| 7 | demethylsuberosin | 230.26 | 3.50 | 3 | 1 | 50.44 | 2 | 2 | 2 | 0.634 | TRUE |
| 8 | decursinol | 246.26 | 2.81 | 4 | 1 | 59.67 | 0 | 2 | 1 | 0.726 | TRUE |
| 9 | xanthoarnol | 262.26 | 1.88 | 5 | 2 | 79.90 | 1 | 2 | 1 | 0.767 | TRUE |
| 10 | marmesin | 246.26 | 2.78 | 4 | 1 | 59.67 | 1 | 2 | 1 | 0.786 | TRUE |
| 11 | peucedanone | 262.26 | 2.45 | 5 | 2 | 87.74 | 3 | 2 | 1 | 0.829 | TRUE |
| 12 | xanthyletin | 228.24 | 3.32 | 3 | 0 | 39.44 | 0 | 3 | 1 | 0.554 | TRUE |
| 13 | Z-ligustilide | 190.24 | 2.99 | 2 | 0 | 26.30 | 2 | 1 | 0 | 0.716 | TRUE |
| 14 | Butylidenephthalide | 188.22 | 3.33 | 2 | 0 | 26.30 | 2 | 1 | 0 | 0.711 | TRUE |
| | | | | | ***Paeonia lactiflora*** | | | | | | |
| 15 | albiflorin | 480.46 | -0.25 | 11 | 5 | 172.21 | 7 | 1 | 1 | 0.326 | FALSE |
| 16 | oleanolic acid | 456.7 | 5.85 | 3 | 2 | 57.53 | 1 | 0 | 1 | 0.487 | TRUE |
| 17 | paeoniflorin | 480.46 | -0.36 | 11 | 5 | 164.37 | 7 | 1 | 0 | 0.346 | FALSE |
| 18 | paeonol | 166.17 | 1.68 | 3 | 1 | 46.53 | 2 | 1 | 0 | 0.727 | TRUE |
| 19 | benzoic acid | 122.12 | 1.24 | 2 | 1 | 37.30 | 1 | 1 | 0 | 0.608 | TRUE |
| 20 | daucosterol | 576.85 | 5.02 | 6 | 4 | 99.38 | 9 | 0 | 1 | 0.288 | TRUE |
| 21 | betulinic acid | 456.7 | 5.75 | 3 | 2 | 57.53 | 2 | 0 | 1 | 0.520 | TRUE |
| 22 | hederagenin | 472.7 | 5.24 | 4 | 3 | 77.76 | 2 | 0 | 1 | 0.480 | TRUE |
| 23 | oxypaeoniflorin | 496.46 | -0.83 | 12 | 6 | 184.60 | 7 | 1 | 0 | 0.276 | FALSE |
| 24 | 4-*O*-methylpaeoniflorin | 494.49 | 0.19 | 11 | 4 | 153.37 | 8 | 1 | 0 | 0.392 | FALSE |
| 25 | $\rho$-hydroxybenzoic acid | 138.12 | 0.74 | 3 | 2 | 57.53 | 1 | 1 | 0 | 0.601 | TRUE |
| 26 | 30-norhederagenin | 456.66 | 5.00 | 4 | 3 | 77.76 | 2 | 0 | 1 | 0.511 | TRUE |
| 27 | mudanpioside E | 526.49 | -0.75 | 13 | 6 | 193.83 | 8 | 1 | 0 | 0.248 | FALSE |
| 28 | lactiflorin | 462.45 | 0.52 | 10 | 3 | 140.98 | 5 | 1 | 0 | 0.567 | FALSE |

| | | | | | | | | | | | |
|---|---|---|---|---|---|---|---|---|---|---|---|
| 29 | 3,3'-di-*O*-methylellagic acid | 330.25 | 2.71 | 8 | 2 | 119.34 | 2 | 4 | 2 | 0.425 | TRUE |
| 30 | mudanpioside C | 600.57 | 0.70 | 13 | 5 | 190.67 | 10 | 2 | 1 | 0.240 | FALSE |
| 31 | paeonidanin A | 598.59 | 1.86 | 12 | 3 | 167.28 | 11 | 2 | 1 | 0.322 | FALSE |
| 32 | paeonidanin B | 646.59 | 0.46 | 15 | 6 | 227.97 | 11 | 2 | 2 | 0.144 | FALSE |
| 33 | paeonidanin C | 614.59 | 1.39 | 13 | 4 | 187.51 | 11 | 2 | 1 | 0.266 | FALSE |
| 34 | paeoniflorigenone | 318.32 | 1.95 | 6 | 1 | 82.06 | 4 | 1 | 0 | 0.914 | TRUE |
| 35 | aromadendrin | 288.25 | 1.15 | 6 | 4 | 107.22 | 1 | 3 | 0 | 0.535 | TRUE |
| 36 | $\beta$-amyrin | 426.72 | 6.92 | 1 | 1 | 20.23 | 0 | 0 | 1 | 0.417 | TRUE |
| 37 | arjunolic acid | 488.7 | 4.35 | 5 | 2 | 97.99 | 0 | 0 | 1 | 0.494 | TRUE |
| 38 | 24-methylenecycloartanol | 440.74 | 7.93 | 1 | 1 | 20.23 | 5 | 0 | 1 | 0.430 | TRUE |
| 39 | palbinone | 358.47 | 3.44 | 4 | 2 | 74.60 | 0 | 0 | 1 | 0.651 | TRUE |
| 40 | benzoylpaeoniflorin | 584.57 | 1.17 | 12 | 4 | 170.44 | 10 | 2 | 1 | 0.295 | FALSE |
| 41 | 8-debenzoylpaeoniflorin | 376.36 | -1.88 | 10 | 6 | 158.30 | 4 | 0 | 0 | 0.328 | FALSE |
| 42 | paeonilactone A | 198.22 | 1.10 | 4 | 1 | 63.60 | 0 | 0 | 0 | 0.633 | TRUE |
| 43 | paeonilactone B | 196.2 | 1.37 | 4 | 1 | 63.60 | 0 | 0 | 0 | 0.635 | TRUE |
| 44 | paeonilactone C | 318.32 | 2.04 | 6 | 1 | 89.90 | 4 | 1 | 1 | 0.854 | TRUE |
| 45 | pyrethrin I | 328.45 | 5.27 | 3 | 0 | 43.37 | 7 | 0 | 2 | 0.472 | TRUE |
| 46 | pyrethrin II | 372.45 | 4.82 | 5 | 0 | 69.67 | 9 | 0 | 3 | 0.330 | TRUE |
| | | | | | ***Lindera strychnifolia*** | | | | | | |
| 47 | linderane | 260.29 | 3.20 | 4 | 0 | 51.97 | 4 | 0 | 2 | 0.575 | TRUE |
| 48 | norisoboldine | 313.35 | 3.02 | 5 | 3 | 70.95 | 2 | 2 | 0 | 0.795 | TRUE |
| 49 | lindestrene | 214.30 | 4.18 | 1 | 0 | 13.14 | 0 | 0 | 1 | 0.551 | TRUE |
| 50 | linderene | 230.30 | 3.25 | 2 | 1 | 33.37 | 0 | 0 | 1 | 0.648 | TRUE |
| 51 | chamazulene | 184.28 | 4.78 | 0 | 0 | 0.00 | 1 | 1 | 0 | 0.589 | TRUE |
| 52 | linderazulene | 210.27 | 4.89 | 1 | 0 | 13.14 | 0 | 1 | 0 | 0.594 | TRUE |
| 53 | linderalactone | 244.29 | 3.34 | 3 | 0 | 39.44 | 0 | 0 | 1 | 0.613 | TRUE |
| 54 | isolinderalactone | 244.29 | 3.81 | 3 | 0 | 39.44 | 1 | 0 | 2 | 0.520 | TRUE |
| 55 | strychnistenolide | 262.30 | 1.81 | 4 | 2 | 66.76 | 0 | 0 | 1 | 0.653 | TRUE |
| 56 | epicatechin | 290.27 | 0.98 | 6 | 5 | 110.38 | 1 | 2 | 1 | 0.501 | TRUE |
| 57 | 6,7-dimethoxy-2-(2-phenylethyl)chromone | 310.34 | 4.80 | 4 | 0 | 48.67 | 5 | 3 | 0 | 0.679 | TRUE |
| 58 | 6,7-Dimethoxy-2-(2-(4-methoxyphenyl)ethyl)chromone | 340.37 | 4.86 | 5 | 0 | 57.90 | 6 | 3 | 0 | 0.641 | TRUE |

| | | | | | | | | | | | |
|---|---|---|---|---|---|---|---|---|---|---|---|
| 59 | norboldine | 313.35 | 3.02 | 5 | 3 | 70.95 | 2 | 2 | 0 | 0.795 | TRUE |
| 60 | nubigenol | 290.27 | 1.40 | 6 | 5 | 118.22 | 4 | 2 | 0 | 0.581 | TRUE |
| 61 | boldine | 327.37 | 2.95 | 5 | 2 | 62.16 | 2 | 2 | 0 | 0.888 | TRUE |
| | | | | | ***Cyperus rotundus*** | | | | | | |
| 62 | cyperene | 204.35 | 4.3 | 0 | 0 | 0.00 | 0 | 0 | 1 | 0.516 | TRUE |
| 63 | $\alpha$-copaene | 204.35 | 3.73 | 0 | 0 | 0.00 | 1 | 0 | 1 | 0.571 | TRUE |
| 64 | $\alpha$-selinene | 204.35 | 4.14 | 0 | 0 | 0.00 | 1 | 0 | 1 | 0.563 | TRUE |
| 65 | rotundene | 204.35 | 3.70 | 0 | 0 | 0.00 | 0 | 0 | 1 | 0.528 | TRUE |
| 66 | valencene | 204.35 | 4.14 | 0 | 0 | 0.00 | 1 | 0 | 1 | 0.563 | TRUE |
| 67 | $\beta$-gurjunene | 204.35 | 3.80 | 0 | 0 | 0.00 | 0 | 0 | 1 | 0.526 | TRUE |
| 68 | trans-calamene | 202.34 | 4.48 | 0 | 0 | 0.00 | 1 | 1 | 0 | 0.630 | TRUE |
| 69 | $\delta$-cadinene | 204.35 | 4.01 | 0 | 0 | 0.00 | 1 | 0 | 1 | 0.566 | TRUE |
| 70 | $\gamma$-calacorene | 200.32 | 4.47 | 0 | 0 | 0.00 | 1 | 1 | 0 | 0.627 | TRUE |
| 71 | epi-$\alpha$-selinene | 204.35 | 4.14 | 0 | 0 | 0.00 | 1 | 0 | 1 | 0.563 | TRUE |
| 72 | $\alpha$-mauurolene | 204.35 | 3.73 | 0 | 0 | 0.00 | 1 | 0 | 0 | 0.610 | TRUE |
| 73 | cadalene | 198.3 | 4.97 | 0 | 0 | 0.00 | 1 | 2 | 0 | 0.607 | TRUE |
| 74 | nootkatene | 202.34 | 3.74 | 0 | 0 | 0.00 | 1 | 0 | 1 | 0.568 | TRUE |
| 75 | cyperotundone | 218.33 | 3.97 | 1 | 0 | 17.07 | 0 | 0 | 0 | 0.601 | TRUE |
| 76 | mustakone | 218.33 | 3.40 | 1 | 0 | 17.07 | 1 | 0 | 0 | 0.659 | TRUE |
| 77 | cyperol | 220.35 | 3.62 | 1 | 1 | 20.23 | 1 | 0 | 1 | 0.669 | TRUE |
| 78 | isocyperol | 220.35 | 3.51 | 1 | 1 | 20.23 | 1 | 0 | 0 | 0.717 | TRUE |
| 79 | $\alpha$-cyperone | 218.33 | 4.21 | 1 | 0 | 17.07 | 1 | 0 | 1 | 0.602 | TRUE |
| 80 | chrysoeriol | 300.26 | 2.55 | 6 | 3 | 100.13 | 2 | 3 | 0 | 0.672 | TRUE |
| 81 | nootkatone | 218.33 | 3.82 | 1 | 0 | 17.07 | 1 | 0 | 1 | 0.611 | TRUE |
| | | | | | ***Caesalpinia sappan*** | | | | | | |
| 82 | sappanchalcone | 286.28 | 2.53 | 5 | 3 | 86.99 | 4 | 2 | 2 | 0.594 | TRUE |
| 83 | 3-deoxysappanone B | 286.28 | 2.42 | 5 | 3 | 86.99 | 2 | 2 | 1 | 0.738 | TRUE |
| 84 | 3-deoxysappanchalcone | 270.28 | 3.00 | 4 | 2 | 66.76 | 4 | 2 | 1 | 0.838 | TRUE |
| 85 | 3′-deoxy-4-*O*-methylepisappanol | 302.32 | 2.33 | 5 | 3 | 79.15 | 3 | 2 | 0 | 0.811 | TRUE |
| 86 | brazilin | 286.28 | 1.86 | 5 | 4 | 90.15 | 0 | 2 | 1 | 0.555 | TRUE |
| 87 | brazilein | 284.26 | 1.68 | 5 | 3 | 86.99 | 0 | 1 | 1 | 0.631 | TRUE |

| | | | | | | | | | | | |
|---|---|---|---|---|---|---|---|---|---|---|---|
| 88 | brazilane | 270.28 | 2.37 | 4 | 3 | 69.92 | 0 | 2 | 1 | 0.643 | TRUE |
| 89 | hematoxyline | 302.28 | 1.37 | 6 | 5 | 110.38 | 0 | 2 | 1 | 0.470 | TRUE |
| 90 | hematein | 300.26 | 1.17 | 6 | 4 | 107.22 | 0 | 1 | 1 | 0.536 | TRUE |
| | | | | ***Catharmus tinctorius*** | | | | | | | |
| 91 | 6-hydroxykaempferol | 302.24 | 1.54 | 7 | 5 | 131.36 | 1 | 3 | 1 | 0.431 | TRUE |
| 92 | carthamone | 448.38 | -0.53 | 11 | 6 | 191.05 | 6 | 1 | 3 | 0.186 | FALSE |
| 93 | quercetagetin | 318.24 | 1.06 | 8 | 6 | 151.59 | 1 | 3 | 1 | 0.364 | FALSE |
| 94 | kaempferol | 286.24 | 2.03 | 6 | 4 | 111.13 | 1 | 3 | 0 | 0.544 | TRUE |
| 95 | luteolin | 286.24 | 2.03 | 6 | 4 | 111.13 | 1 | 3 | 1 | 0.509 | TRUE |
| 96 | quercetin | 302.24 | 1.54 | 7 | 5 | 131.36 | 1 | 3 | 1 | 0.431 | TRUE |
| 97 | apigenin | 270.24 | 2.52 | 5 | 3 | 90.90 | 1 | 3 | 0 | 0.632 | TRUE |
| 98 | safflomin A | 612.53 | -3.25 | 16 | 12 | 295.36 | 6 | 1 | 3 | 0.085 | FALSE |
| 99 | sophoricoside | 432.38 | 0.35 | 10 | 6 | 170.05 | 4 | 3 | 0 | 0.338 | FALSE |
| | | | | ***Prunus persica*** | | | | | | | |
| 100 | amygdalin | 457.43 | -2.71 | 12 | 7 | 202.32 | 7 | 1 | 0 | 0.223 | FALSE |
| 101 | prunasin | 295.29 | -0.66 | 7 | 4 | 123.17 | 4 | 1 | 0 | 0.581 | TRUE |
| 102 | neoamygdalin | 457.43 | -2.71 | 12 | 7 | 202.32 | 7 | 1 | 0 | 0.223 | FALSE |
| 103 | vanilloside | 316.30 | -0.80 | 8 | 5 | 128.84 | 5 | 1 | 0 | 0.477 | FALSE |
| 104 | lacticolorin | 406.38 | 0.22 | 9 | 5 | 145.91 | 7 | 2 | 0 | 0.436 | FALSE |
| | | | | ***Cinnamomum cassia*** | | | | | | | |
| 105 | cinnamic acid | 148.16 | 1.70 | 2 | 1 | 37.30 | 2 | 1 | 1 | 0.648 | TRUE |
| 106 | cinnamaldehyde | 132.16 | 2.48 | 1 | 0 | 17.07 | 2 | 1 | 2 | 0.446 | TRUE |
| 107 | coumarin | 146.14 | 2.50 | 2 | 0 | 30.21 | 0 | 1 | 1 | 0.526 | TRUE |
| | | | | ***Glycyrrhiza uralenesis*** | | | | | | | |
| 108 | glycyrrhizin | 822.93 | 0.52 | 16 | 8 | 267.04 | 7 | 0 | 1 | 0.163 | FALSE |
| 109 | liquiritin | 418.39 | 0.36 | 9 | 5 | 145.91 | 4 | 2 | 0 | 0.475 | FALSE |
| 110 | liquiritigenin | 256.25 | 2.55 | 4 | 2 | 66.76 | 1 | 2 | 0 | 0.822 | TRUE |
| 111 | isoliquiritin | 418.39 | 0.42 | 9 | 6 | 156.91 | 6 | 2 | 1 | 0.366 | FALSE |
| 112 | isoliquiritigenin | 256.25 | 2.48 | 4 | 3 | 77.76 | 3 | 2 | 1 | 0.737 | TRUE |
| 113 | formonetin | 284.26 | 3.03 | 5 | 2 | 79.9 | 2 | 3 | 1 | 0.707 | TRUE |
| 114 | ononin | 430.40 | 1.36 | 9 | 4 | 138.82 | 5 | 3 | 0 | 0.472 | FALSE |
| 115 | glycycoumarin | 368.38 | 4.28 | 6 | 3 | 100.13 | 4 | 2 | 2 | 0.550 | TRUE |

| | | | | | | | | | | | |
|---|---|---|---|---|---|---|---|---|---|---|---|
| 116 | glycyrol | 366.36 | 4.62 | 6 | 2 | 93.04 | 3 | 2 | 2 | 0.608 | TRUE |
| 117 | prunetin | 284.26 | 3.03 | 5 | 2 | 79.9 | 2 | 3 | 0 | 0.756 | TRUE |
| 118 | glycyrrhetinic acid | 470.68 | 5.55 | 4 | 2 | 74.6 | 1 | 0 | 0 | 0.527 | TRUE |
| 119 | licoricidin | 424.53 | 5.93 | 5 | 3 | 79.15 | 6 | 2 | 1 | 0.501 | TRUE |

Table S2. List of expected active compounds

| No. | Compound Name | MW | ALOGP | HBA | HBD | PSA | ROTB | AROM | ALERTS | QED | OB |
|---|---|---|---|---|---|---|---|---|---|---|---|
| | | | | | *Angelica gigas* | | | | | | |
| 1 | decursin | 328.36 | 4.06 | 5 | 0 | 65.74 | 3 | 2 | 2 | 0.628 | TRUE |
| 2 | decursinol angelate | 328.36 | 4.06 | 5 | 0 | 65.74 | 3 | 2 | 2 | 0.628 | TRUE |
| 3 | isoimperatorin | 270.29 | 3.99 | 4 | 0 | 52.58 | 3 | 2 | 2 | 0.625 | TRUE |
| 4 | ferulic acid | 194.18 | 1.26 | 4 | 2 | 66.76 | 3 | 1 | 1 | 0.711 | TRUE |
| 5 | umbelliferone | 162.14 | 1.97 | 3 | 1 | 50.44 | 0 | 2 | 1 | 0.602 | TRUE |
| 6 | demethylsuberosin | 230.26 | 3.50 | 3 | 1 | 50.44 | 2 | 2 | 2 | 0.634 | TRUE |
| 7 | decursinol | 246.26 | 2.81 | 4 | 1 | 59.67 | 0 | 2 | 1 | 0.726 | TRUE |
| 8 | xanthoarnol | 262.26 | 1.88 | 5 | 2 | 79.90 | 1 | 2 | 1 | 0.767 | TRUE |
| 9 | marmesin | 246.26 | 2.78 | 4 | 1 | 59.67 | 1 | 2 | 1 | 0.786 | TRUE |
| 10 | peucedanone | 262.26 | 2.45 | 5 | 2 | 87.74 | 3 | 2 | 1 | 0.829 | TRUE |
| 11 | xanthyletin | 228.24 | 3.32 | 3 | 0 | 39.44 | 0 | 3 | 1 | 0.554 | TRUE |
| 12 | Z-ligustilide | 190.24 | 2.99 | 2 | 0 | 26.30 | 2 | 1 | 0 | 0.716 | TRUE |
| 13 | Butylidenephthalide | 188.22 | 3.33 | 2 | 0 | 26.30 | 2 | 1 | 0 | 0.711 | TRUE |
| | | | | | *Paeonia lactiflora* | | | | | | |
| 14 | oleanolic acid | 456.7 | 5.85 | 3 | 2 | 57.53 | 1 | 0 | 1 | 0.487 | TRUE |
| 15 | paeonol | 166.17 | 1.68 | 3 | 1 | 46.53 | 2 | 1 | 0 | 0.727 | TRUE |
| 16 | benzoic acid | 122.12 | 1.24 | 2 | 1 | 37.30 | 1 | 1 | 0 | 0.608 | TRUE |
| 17 | daucosterol | 576.85 | 5.02 | 6 | 4 | 99.38 | 9 | 0 | 1 | 0.288 | TRUE |
| 18 | betulinic acid | 456.7 | 5.75 | 3 | 2 | 57.53 | 2 | 0 | 1 | 0.520 | TRUE |
| 19 | hederagenin | 472.7 | 5.24 | 4 | 3 | 77.76 | 2 | 0 | 1 | 0.480 | TRUE |
| 20 | $\rho$-hydroxybenzoic acid | 138.12 | 0.74 | 3 | 2 | 57.53 | 1 | 1 | 0 | 0.601 | TRUE |
| 21 | 30-norhederagenin | 456.66 | 5.00 | 4 | 3 | 77.76 | 2 | 0 | 1 | 0.511 | TRUE |
| 22 | 3,3'-di-*O*-methylellagic acid | 330.25 | 2.71 | 8 | 2 | 119.34 | 2 | 4 | 2 | 0.425 | TRUE |
| 23 | paeoniflorigenone | 318.32 | 1.95 | 6 | 1 | 82.06 | 4 | 1 | 0 | 0.914 | TRUE |
| 24 | aromadendrin | 288.25 | 1.15 | 6 | 4 | 107.22 | 1 | 3 | 0 | 0.535 | TRUE |
| 25 | $\beta$-amyrin | 426.72 | 6.92 | 1 | 1 | 20.23 | 0 | 0 | 1 | 0.417 | TRUE |
| 26 | arjunolic acid | 488.7 | 4.35 | 5 | 2 | 97.99 | 0 | 0 | 1 | 0.494 | TRUE |
| 27 | 24-methylenecycloartanol | 440.74 | 7.93 | 1 | 1 | 20.23 | 5 | 0 | 1 | 0.430 | TRUE |

| | | | | | | | | | | | |
|---|---|---|---|---|---|---|---|---|---|---|---|
| 28 | palbinone | 358.47 | 3.44 | 4 | 2 | 74.60 | 0 | 0 | 1 | 0.651 | TRUE |
| 29 | paeonilactone A | 198.22 | 1.10 | 4 | 1 | 63.60 | 0 | 0 | 0 | 0.633 | TRUE |
| 30 | paeonilactone B | 196.2 | 1.37 | 4 | 1 | 63.60 | 0 | 0 | 0 | 0.635 | TRUE |
| 31 | paeonilactone C | 318.32 | 2.04 | 6 | 1 | 89.90 | 4 | 1 | 1 | 0.854 | TRUE |
| 32 | pyrethrin I | 328.45 | 5.27 | 3 | 0 | 43.37 | 7 | 0 | 2 | 0.472 | TRUE |
| 33 | pyrethrin II | 372.45 | 4.82 | 5 | 0 | 69.67 | 9 | 0 | 3 | 0.330 | TRUE |
| | | | | | ***Lindera strychnifolin*** | | | | | | |
| 34 | linderane | 260.29 | 3.20 | 4 | 0 | 51.97 | 4 | 0 | 2 | 0.575 | TRUE |
| 35 | norisoboldine | 313.35 | 3.02 | 5 | 3 | 70.95 | 2 | 2 | 0 | 0.795 | TRUE |
| 36 | lindestrene | 214.30 | 4.18 | 1 | 0 | 13.14 | 0 | 0 | 1 | 0.551 | TRUE |
| 37 | linderene | 230.30 | 3.25 | 2 | 1 | 33.37 | 0 | 0 | 1 | 0.648 | TRUE |
| 38 | chamazulene | 184.28 | 4.78 | 0 | 0 | 0.00 | 1 | 1 | 0 | 0.589 | TRUE |
| 39 | linderazulene | 210.27 | 4.89 | 1 | 0 | 13.14 | 0 | 1 | 0 | 0.594 | TRUE |
| 40 | linderalactone | 244.29 | 3.34 | 3 | 0 | 39.44 | 0 | 0 | 1 | 0.613 | TRUE |
| 41 | isolinderalactone | 244.29 | 3.81 | 3 | 0 | 39.44 | 1 | 0 | 2 | 0.520 | TRUE |
| 42 | strychnistenolide | 262.30 | 1.81 | 4 | 2 | 66.76 | 0 | 0 | 1 | 0.653 | TRUE |
| 43 | epicatechin | 290.27 | 0.98 | 6 | 5 | 110.38 | 1 | 2 | 1 | 0.501 | TRUE |
| 44 | 6,7-dimethoxy-2-(2-phenylethyl)chromone | 310.34 | 4.80 | 4 | 0 | 48.67 | 5 | 3 | 0 | 0.679 | TRUE |
| 45 | 6,7-Dimethoxy-2-(2-(4-methoxyphenyl)ethyl)chromone | 340.37 | 4.86 | 5 | 0 | 57.90 | 6 | 3 | 0 | 0.641 | TRUE |
| 46 | norboldine | 313.35 | 3.02 | 5 | 3 | 70.95 | 2 | 2 | 0 | 0.795 | TRUE |
| 47 | nubigenol | 290.27 | 1.40 | 6 | 5 | 118.22 | 4 | 2 | 0 | 0.581 | TRUE |
| 48 | boldine | 327.37 | 2.95 | 5 | 2 | 62.16 | 2 | 2 | 0 | 0.888 | TRUE |
| | | | | | ***Cyperus rotundus*** | | | | | | |
| 49 | cyperene | 204.35 | 4.3 | 0 | 0 | 0.00 | 0 | 0 | 1 | 0.516 | TRUE |
| 50 | $\alpha$-copaene | 204.35 | 3.73 | 0 | 0 | 0.00 | 1 | 0 | 1 | 0.571 | TRUE |
| 51 | $\alpha$-selinene | 204.35 | 4.14 | 0 | 0 | 0.00 | 1 | 0 | 1 | 0.563 | TRUE |
| 52 | rotundene | 204.35 | 3.70 | 0 | 0 | 0.00 | 0 | 0 | 1 | 0.528 | TRUE |
| 53 | valencene | 204.35 | 4.14 | 0 | 0 | 0.00 | 1 | 0 | 1 | 0.563 | TRUE |
| 54 | $\beta$-gurjunene | 204.35 | 3.80 | 0 | 0 | 0.00 | 0 | 0 | 1 | 0.526 | TRUE |
| 55 | trans-calamene | 202.34 | 4.48 | 0 | 0 | 0.00 | 1 | 1 | 0 | 0.630 | TRUE |
| 56 | $\delta$-cadinene | 204.35 | 4.01 | 0 | 0 | 0.00 | 1 | 0 | 1 | 0.566 | TRUE |

| | | | | | | | | | | | |
|---|---|---|---|---|---|---|---|---|---|---|---|
| 57 | $\gamma$-calacorene | 200.32 | 4.47 | 0 | 0 | 0.00 | 1 | 1 | 0 | 0.627 | TRUE |
| 58 | epi-$\alpha$-selinene | 204.35 | 4.14 | 0 | 0 | 0.00 | 1 | 0 | 1 | 0.563 | TRUE |
| 59 | $\alpha$-mauurolene | 204.35 | 3.73 | 0 | 0 | 0.00 | 1 | 0 | 0 | 0.610 | TRUE |
| 60 | cadalene | 198.3 | 4.97 | 0 | 0 | 0.00 | 1 | 2 | 0 | 0.607 | TRUE |
| 61 | nootkatene | 202.34 | 3.74 | 0 | 0 | 0.00 | 1 | 0 | 1 | 0.568 | TRUE |
| 62 | cyperotundone | 218.33 | 3.97 | 1 | 0 | 17.07 | 0 | 0 | 0 | 0.601 | TRUE |
| 63 | mustakone | 218.33 | 3.40 | 1 | 0 | 17.07 | 1 | 0 | 0 | 0.659 | TRUE |
| 64 | cyperol | 220.35 | 3.62 | 1 | 1 | 20.23 | 1 | 0 | 1 | 0.669 | TRUE |
| 65 | isocyperol | 220.35 | 3.51 | 1 | 1 | 20.23 | 1 | 0 | 0 | 0.717 | TRUE |
| 66 | $\alpha$-cyperone | 218.33 | 4.21 | 1 | 0 | 17.07 | 1 | 0 | 1 | 0.602 | TRUE |
| 67 | chrysoeriol | 300.26 | 2.55 | 6 | 3 | 100.13 | 2 | 3 | 0 | 0.672 | TRUE |
| 68 | nootkatone | 218.33 | 3.82 | 1 | 0 | 17.07 | 1 | 0 | 1 | 0.611 | TRUE |
| | | | | | ***Caesalpinia sappan*** | | | | | | |
| 69 | sappanchalcone | 286.28 | 2.53 | 5 | 3 | 86.99 | 4 | 2 | 2 | 0.594 | TRUE |
| 70 | 3-deoxysappanone B | 286.28 | 2.42 | 5 | 3 | 86.99 | 2 | 2 | 1 | 0.738 | TRUE |
| 71 | 3-deoxysappanchalcone | 270.28 | 3.00 | 4 | 2 | 66.76 | 4 | 2 | 1 | 0.838 | TRUE |
| 72 | 3′-deoxy-4-*O*-methylepisappanol | 302.32 | 2.33 | 5 | 3 | 79.15 | 3 | 2 | 0 | 0.811 | TRUE |
| 73 | brazilin | 286.28 | 1.86 | 5 | 4 | 90.15 | 0 | 2 | 1 | 0.555 | TRUE |
| 74 | brazilein | 284.26 | 1.68 | 5 | 3 | 86.99 | 0 | 1 | 1 | 0.631 | TRUE |
| 75 | brazilane | 270.28 | 2.37 | 4 | 3 | 69.92 | 0 | 2 | 1 | 0.643 | TRUE |
| 76 | hematoxyline | 302.28 | 1.37 | 6 | 5 | 110.38 | 0 | 2 | 1 | 0.470 | TRUE |
| 77 | hematein | 300.26 | 1.17 | 6 | 4 | 107.22 | 0 | 1 | 1 | 0.536 | TRUE |
| | | | | | ***Catharmus tinctorius*** | | | | | | |
| 78 | 6-hydroxykaempferol | 302.24 | 1.54 | 7 | 5 | 131.36 | 1 | 3 | 1 | 0.431 | TRUE |
| 79 | kaempferol | 286.24 | 2.03 | 6 | 4 | 111.13 | 1 | 3 | 0 | 0.544 | TRUE |
| 80 | luteolin | 286.24 | 2.03 | 6 | 4 | 111.13 | 1 | 3 | 1 | 0.509 | TRUE |
| 81 | quercetin | 302.24 | 1.54 | 7 | 5 | 131.36 | 1 | 3 | 1 | 0.431 | TRUE |
| 82 | apigenin | 270.24 | 2.52 | 5 | 3 | 90.90 | 1 | 3 | 0 | 0.632 | TRUE |
| | | | | | ***Prunus persica*** | | | | | | |
| 83 | prunasin | 295.29 | -0.66 | 7 | 4 | 123.17 | 4 | 1 | 0 | 0.581 | TRUE |
| | | | | | ***Cinnamomum cassia*** | | | | | | |

| | | | | | | | | | | | |
|---|---|---|---|---|---|---|---|---|---|---|---|
| 84 | cinnamic acid | 148.16 | 1.70 | 2 | 1 | 37.30 | 2 | 1 | 1 | 0.648 | TRUE |
| 85 | cinnamaldehyde | 132.16 | 2.48 | 1 | 0 | 17.07 | 2 | 1 | 2 | 0.446 | TRUE |
| 86 | coumarin | 146.14 | 2.50 | 2 | 0 | 30.21 | 0 | 1 | 1 | 0.526 | TRUE |
| | | | | | ***Glycyrrhiza uralenesis*** | | | | | | |
| 87 | liquiritigenin | 256.25 | 2.55 | 4 | 2 | 66.76 | 1 | 2 | 0 | 0.822 | TRUE |
| 88 | isoliquiritigenin | 256.25 | 2.48 | 4 | 3 | 77.76 | 3 | 2 | 1 | 0.737 | TRUE |
| 89 | formonetin | 284.26 | 3.03 | 5 | 2 | 79.9 | 2 | 3 | 1 | 0.707 | TRUE |
| 90 | glycycoumarin | 368.38 | 4.28 | 6 | 3 | 100.13 | 4 | 2 | 2 | 0.550 | TRUE |
| 91 | glycyrol | 366.36 | 4.62 | 6 | 2 | 93.04 | 3 | 2 | 2 | 0.608 | TRUE |
| 92 | prunetin | 284.26 | 3.03 | 5 | 2 | 79.9 | 2 | 3 | 0 | 0.756 | TRUE |
| 93 | glycyrrhetinic acid | 470.68 | 5.55 | 4 | 2 | 74.6 | 1 | 0 | 0 | 0.527 | TRUE |
| 94 | licoricidin | 424.53 | 5.93 | 5 | 3 | 79.15 | 6 | 2 | 1 | 0.501 | TRUE |

Table S3. List of potential targets

| No. | Uniprot ID | Gene | Relevance score | Protein class |
|---|---|---|---|---|
| 1 | P00734 | F2 | 58.855 | Enzyme |
| 2 | O60674 | JAK2 | 36.781 | Kinase |
| 3 | P13726 | F3 | 23.179 | Receptor |
| 4 | P05121 | SERPINE1 | 21.828 | Enzyme modulator |
| 5 | P00742 | F10 | 15.713 | Enzyme |
| 6 | P00747 | PLG | 12.016 | Enzyme |
| 7 | P31749 | AKT1 | 11.390 | Kinase |
| 8 | P42336 | PIK3CA | 9.507 | Kinase |
| 9 | P11712 | CYP2C9 | 8.860 | - |
| 10 | P05231 | IL6 | 8.536 | - |
| 11 | P37023 | ACVRL1 | 8.031 | Kinase |
| 12 | P29474 | NOS3 | 7.956 | - |
| 13 | P15692 | VEGFA | 7.895 | Signaling |
| 14 | P16581 | SELE | 7.715 | - |
| 15 | P01911 | HLA-DRB1 | 7.697 | Immune response |
| 16 | O00206 | TLR4 | 7.630 | - |
| 17 | P02768 | ALB | 7.226 | Transporter |
| 18 | P01375 | TNF | 7.125 | Signaling |
| 19 | Q13315 | ATM | 6.941 | Kinase |
| 20 | P12821 | ACE | 6.867 | Enzyme |
| 21 | P35354 | PTGS2 | 6.734 | Enzyme |
| 22 | O75874 | IDH1 | 6.726 | - |
| 23 | P33261 | CYP2C19 | 6.485 | - |
| 24 | Q9Y2R2 | PTPN22 | 6.229 | - |
| 25 | P16234 | PDGFRA | 5.720 | Kinase |
| 26 | Q13093 | PLA2G7 | 5.527 | Enzyme |
| 27 | P23219 | PTGS1 | 5.516 | Enzyme |
| 28 | O14746 | TERT | 5.463 | Enzyme |
| 29 | P05362 | ICAM1 | 5.260 | - |
| 30 | P13569 | CFTR | 5.085 | Ion channel |
| 31 | Q08499 | PDE4D | 4.963 | - |
| 32 | Q99895 | CTRC | 4.855 | - |
| 33 | P15085 | CPA1 | 4.744 | Enzyme |
| 34 | P07477 | PRSS1 | 4.744 | Enzyme |
| 35 | P19438 | TNFRSF1A | 4.729 | - |
| 36 | P00749 | PLAU | 4.719 | Enzyme |
| 37 | P19320 | VCAM1 | 4.597 | - |
| 38 | P27169 | PON1 | 4.476 | - |
| 39 | P08581 | MET | 4.397 | Kinase |
| 40 | P32246 | CCR1 | 4.339 | G-protein coupled receptor |
| 41 | P01042 | KNG1 | 4.289 | Enzyme modulator |
| 42 | P34949 | MPI | 4.280 | Enzyme |
| 43 | P05164 | MPO | 4.240 | Enzyme |
| 44 | P35222 | CTNNB1 | 4.002 | - |
| 45 | P08183 | ABCB1 | 3.940 | Transporter |
| 46 | P14780 | MMP9 | 3.936 | Enzyme |
| 47 | P40763 | STAT3 | 3.767 | Nucleic acid binding |

| | | | | |
|---|---|---|---|---|
| 48 | P31645 | SLC6A4 | 3.663 | Transporter |
| 49 | P11509 | CYP2A6 | 3.460 | - |
| 50 | P08254 | MMP3 | 3.432 | Enzyme |
| 51 | P03372 | ESR1 | 3.424 | Nuclear receptor |
| 52 | P09038 | FGF2 | 3.380 | Signaling |
| 53 | P48736 | PIK3CG | 3.213 | Kinase |
| 54 | P08253 | MMP2 | 3.102 | Enzyme |
| 55 | P08684 | CYP3A4 | 3.073 | Enzyme |
| 56 | P04278 | SHBG | 3.028 | - |
| 57 | P60568 | IL2 | 2.973 | - |
| 58 | P52732 | KIF11 | 2.861 | Cellular structure |
| 59 | Q16665 | HIF1A | 2.834 | Transcription factor |
| 60 | P12931 | SRC | 2.834 | Kinase |
| 61 | P05107 | ITGB2 | 2.812 | Receptor |
| 62 | P61073 | CXCR4 | 2.595 | G-protein coupled receptor |
| 63 | Q07817 | BCL2L1 | 2.575 | Signaling |
| 64 | P03956 | MMP1 | 2.561 | Enzyme |
| 65 | P45379 | TNNT2 | 2.557 | Cellular structure |
| 66 | Q9NR96 | TLR9 | 2.523 | - |
| 67 | P37231 | PPARG | 2.515 | Nuclear receptor |
| 68 | P23526 | AHCY | 2.489 | Enzyme |
| 69 | Q06124 | PTPN11 | 2.467 | - |
| 70 | P08246 | ELANE | 2.460 | Enzyme |
| 71 | P19429 | TNNI3 | 2.443 | Cellular structure |
| 72 | P10721 | KIT | 2.435 | Kinase |
| 73 | P04035 | HMGCR | 2.400 | - |
| 74 | Q14790 | CASP8 | 2.362 | Enzyme |
| 75 | P11597 | CETP | 2.356 | - |
| 76 | P00519 | ABL1 | 2.346 | Kinase |
| 77 | P21964 | COMT | 2.320 | Enzyme |
| 78 | P20701 | ITGAL | 2.274 | - |
| 79 | P05067 | APP | 2.270 | Enzyme modulator |
| 80 | Q15910 | EZH2 | 2.228 | Epigenetic regulator |
| 81 | P04818 | TYMS | 2.218 | Enzyme |
| 82 | P42345 | MTOR | 2.209 | Kinase |
| 83 | Q9Y243 | AKT3 | 2.209 | Kinase |
| 84 | P02766 | TTR | 2.190 | Transporter |
| 85 | P14174 | MIF | 2.184 | - |
| 86 | P35968 | KDR | 2.177 | Kinase |
| 87 | Q9GZT9 | EGLN1 | 2.160 | - |
| 88 | P51451 | BLK | 2.154 | Kinase |
| 89 | P09619 | PDGFRB | 2.117 | Kinase |
| 90 | P43405 | SYK | 2.066 | Kinase |
| 91 | P19838 | NFKB1 | 2.011 | Transcription factor |
| 92 | Q99835 | SMO | 1.998 | G-protein coupled receptor |
| 93 | Q14524 | SCN5A | 1.956 | Ion channel |
| 94 | P00533 | EGFR | 1.950 | Kinase |
| 95 | P01130 | LDLR | 1.928 | - |
| 96 | P28223 | HTR2A | 1.883 | G-protein coupled receptor |
